# Cbl promotes MyD88 ubiquitin-mediated degradation in macrophages via phosphorylation and calcium mobilization

**DOI:** 10.3389/fimmu.2025.1679035

**Published:** 2026-01-05

**Authors:** Yan Zhang, Dan Wang, Jie Huang, Zhefan Wang, Jinren Zhu, Feihan Lyu, Zhengyu Jiang

**Affiliations:** 1 Faculty of Anesthesiology, Changhai Hospital, Naval Medical University, Shanghai, China; 2 Department of Gynecology and Obstetrics, Changzheng Hospital, Naval Medical University, Shanghai, China; 3 Yangpu Senior High School, Shanghai, China; 4 High School Affiliated to Fudan University, Shanghai, China; 5 No.1 High School Affiliated to East China Normal University, Shanghai, China; 6 Department of Anesthesiology, Naval Medical Center, Naval Medical University, Shanghai, China

**Keywords:** Cbl, MyD88, toll-like receptor, ubiquitination, macrophage

## Abstract

**Background:**

Precise regulation of Toll-like receptor (TLR) signaling via myeloid differentiation factor 88 (MyD88) is critical for balancing immune defense and inflammation. While ubiquitination represents a dominant mechanism controlling MyD88 stability, the full spectrum of E3 ligases that regulate MyD88 stability remains undefined.

**Methods:**

In the present study, we investigated the regulatory role of Cbl in MyD88 signaling in macrophage.

**Results:**

We identified Cbl as a direct negative regulator of MyD88 in macrophages. Cbl is upregulated during inflammatory responses, and myeloid-specific Cbl deficiency exacerbates cytokine production and MyD88-dependent signaling in macrophages. Mechanistically, Cbl could interact with MyD88. The overexpression of Cbl promoted the ubiquitination and proteasomal degradation of MyD88, whereas Cbl deficiency abrogated its ubiquitination. Moreover, we found that Cbl activation required intracellular calcium and phosphorylation. Calcium chelation and inhibited phosphorylation of Cbl abrogated Cbl-mediated MyD88 downregulation.

**Conclusion:**

findings establish Cbl as a novel suppressor of inflammation and direct-interacting E3 ligase of MyD88 in macrophages. Our study expands the knowledge of intrinsic immunoregulatory networks of TLR– MyD88 signaling and the regulatory mechanism of Cbl, suggesting new therapeutic strategies for inflammatory disorders.

## Introduction

Precise modulation of innate inflammatory signaling is imperative for sustaining immune homeostasis and preventing pathological inflammation (1). Toll-like receptors (TLRs) function as primary sentinels for detecting microbial invasion and tissue damage, triggering potent proinflammatory cascades (2, 3). Myeloid differentiation factor 88 (MyD88) serves as an indispensable adaptor for most TLRs, participating in the formation of the myddosome complex to initiate rapid NF-κB and MAPK activation, which is essential for antimicrobial defense (3, 4). Although multilayered regulation has been revealed for the intrinsic regulation of MyD88, including transcriptional and translational regulation, ubiquitination has emerged as the dominant mechanism dynamically calibrating MyD88 signal amplitude and termination (5–8). Several studies have shown that Smurf, Cbl-b, and Spop suppress MyD88 through K48-linked proteasome degradation (7, 9, 10). Nevertheless, whether additional E3 ligases participate in this regulatory network remains unexplored. Elucidating the ubiquitin-dependent control of MyD88 could provide dynamic regulatory insights and therapeutic targets for inflammatory disorders.

The E3 ubiquitin ligase Cbl (Casitas B-lineage lymphoma), which contains a TKB domain and a RING domain, has been reported to participate in immune regulation in dendritic cells (DCs) (11). The Cbl family contains three members, Cbl, Cbl-b and Cbl-c, among which Cbl and Cbl-b are expressed in immune cells (12). Several studies have revealed that Cbl-b is involved in immune regulation, including suppressing the activation of NK and T cells and promoting MyD88 degradation through the recruitment of Nedd4, a calcium-dependent ubiquitin E3 ligase (10, 13–15). Recent studies have reported that Cbl promotes the ubiquitination and degradation of RelB, a transcription factor involved in antifungal immunity, and enhances the transcription of IL-10, thus suppressing inflammatory responses (16, 17). However, few studies have addressed the direct regulation and mechanism of Cbl in TLR-MyD88 signaling, and its regulatory role in macrophages remains largely unknown.

In the present study, we demonstrated a direct regulatory mechanism of Cbl in MyD88 in macrophages. We showed that Cbl was upregulated during inflammation in macrophages. Myeloid deficiency of Cbl resulted in exacerbated inflammatory cytokine production and upregulation of MyD88 signaling. The overexpression of Cbl promoted MyD88 degradation through the ubiquitin–proteasome pathway, whereas Cbl deficiency abrogated its ubiquitination. Moreover, we found that Cbl activation relies on both intracellular calcium and phosphorylation. Our data revealed a novel role of Cbl in inflammatory responses and specific regulation of MyD88. Our study also provides valuable information concerning the intrinsic regulatory network of MyD88, identifying novel therapeutic targets for inflammatory diseases.

## Materials and methods

### Animal models

Cbl^flox/flox^LysM^Cre+^ (8–10 weeks old; Modelorg Inc., Shanghai) were generated by crossing Cblfl/fl mice with LysM-Cre mice on a C57BL/6J background. In this study, the wild-type (WT) Cbl^flox/flox^LysM^Cre+^ mice used were littermate controls. C57BL/6J mice (8–10 weeks old; Modelorg Inc., Shanghai), Cbl^flox/flox^LysM^Cre+^ and WT mice were maintained under pathogen-free conditions at 18–22°C and 50–60% humidity with a 12 h light/dark cycle and provided ad libitum access to food and water. All procedures were approved by Changhai Hospital’s Ethics Committee following Naval Medical University’s animal regulations.

For acute lung injury (ALI) induction, the mice were anesthetized with 1.5–2% sevoflurane (Drager, Germany) in oxygen. Lipopolysaccharide (LPS; *E. coli* O111:B4, Sigma) in PBS (10 mg/kg, 50 µl) was administered via tracheal intubation. Blood and lung tissues were harvested at 6 h post-instillation. To induce peritonitis in mice, lipopolysaccharide (LPS) (*Escherichia coli* O111:B4, Sigma, China) (10 or 40 mg/kg) diluted in 500 µl of phosphate-buffered saline (PBS) was intraperitoneally injected.

### Clinical sample collection

The collection of human samples was approved by the Ethics Committee of Changhai Hospital, Naval Medical University, and written informed consent was obtained from all volunteers. PBMCs were isolated via standard density gradient centrifugation with Ficoll (Sigma, USA) and cultured in RPMI-1640 (HyClone, USA) for further experiments.

### Bone marrow-derived macrophage culture

Murine femurs were flushed with 3 ml of saline to extract the bone marrow. After RBC lysis, the cells were resuspended (2–4×10^6^ cells/ml) in DMEM supplemented with 10% FBS and 30 ng/ml GM-CSF. Cultures were replenished with fresh medium every 48 h. Mature macrophages were obtained after 5–6 days.

### Cytokine quantification

ELISA (R&D Systems, USA) was used to measure cytokine concentrations in serum/cell supernatants following the manufacturer’s protocols.

### Protein analysis

#### Immunoblotting

The cells were lysed in RIPA buffer (Beyotime, China). Protein concentrations were determined via a BCA assay (Thermo). The samples (10–20 µg) were subjected to SDS–PAGE and transferred to PVDF membranes (Merck, Germany). Blocking was performed with 5% nonfat milk in PBST (pH 7.5) before incubation with primary antibodies (antibody dilution: 1:1500) and HRP-conjugated secondary antibodies (antibody dilution: 1:3000) (Cell Signaling Technology). Protein detection was performed with enhanced chemiluminescence (ECL) reagent (Pierce) and ChemiDoc XRS+ imaging (Bio-Rad). Western blot quantification was performed through calculating target protein gray values/housekeeping protein gray values. The results were defined as relative protein expression. The antibodies used are listed in **Supplementary Table 1**.

#### Immunoprecipitation

Lysates prepared in PIPES buffer (20 mM [pH 6.8], 1% Triton X-100, 150 mM NaCl, 150 mM sucrose, 0.2% sodium deoxycholate, 500 μM EDTA) supplemented with protease inhibitors were centrifuged (14,000 ×g, 10 min). The supernatants were diluted to 2 μg/mL in PIPES dilution buffer (supplemented with 2.5 mM MgCl_2_ and MnCl_2_). After incubation with antibody-bound protein A beads (Sigma) (dilution: 1:1000) at 4°C for 2 h, immunoblotting was performed as described previously.

### DNA transfection

The wild-type and mutant overexpression plasmids were obtained from Obio, China. RAW264.7 cells were cultured in DMEM supplemented with 10% fetal bovine serum (FBS). Before transfection, 4 μg of plasmid was mixed with the jetPEI transfection reagent (Polyplus, France). The resulting mixture was then added to the culture medium for a 24-hour transfection period. After 24 hours, the medium was replaced, and the cells were subjected to subsequent experiments.

### siRNA transfection

BMDMs were seeded in 50% FBS-free medium and transfected with 3 ng/mL siRNA (or control vector) via INTERFEREin (Invitrogen). After 6 h, complete medium was added. The cells were analyzed 48 h post-transfection (**Supplementary Table 1**: siRNA sequences).

### Quantitative PCR

Total RNA isolated with TRIzol (Thermo Fisher) was reverse transcribed via oligo(dT) primers and RT premix (Takara, Japan). qPCR was performed with SYBR Green Master Mix (Takara) on an ABI 7500 cycler (Thermo Fisher): 95°C × 3 min → 40 cycles of 95°C × 10 s → 60°C × 5 s → 72 °C × 10 s. The mRNA levels were normalized to those of *B2m* (**Supplementary Table 2**: primers).

### Statistical analysis

The obtained results were subjected to statistical analysis via Student’s t test or analysis of variance (two-way ANOVA) with appropriate multiple comparisons, as applicable, via Prism software (version 10.0; GraphPad Software, Inc.). A *p* value less than 0.05 was considered statistically significant.

## Results

### Cbl is upregulated in macrophages during inflammation

**Figure 1:**
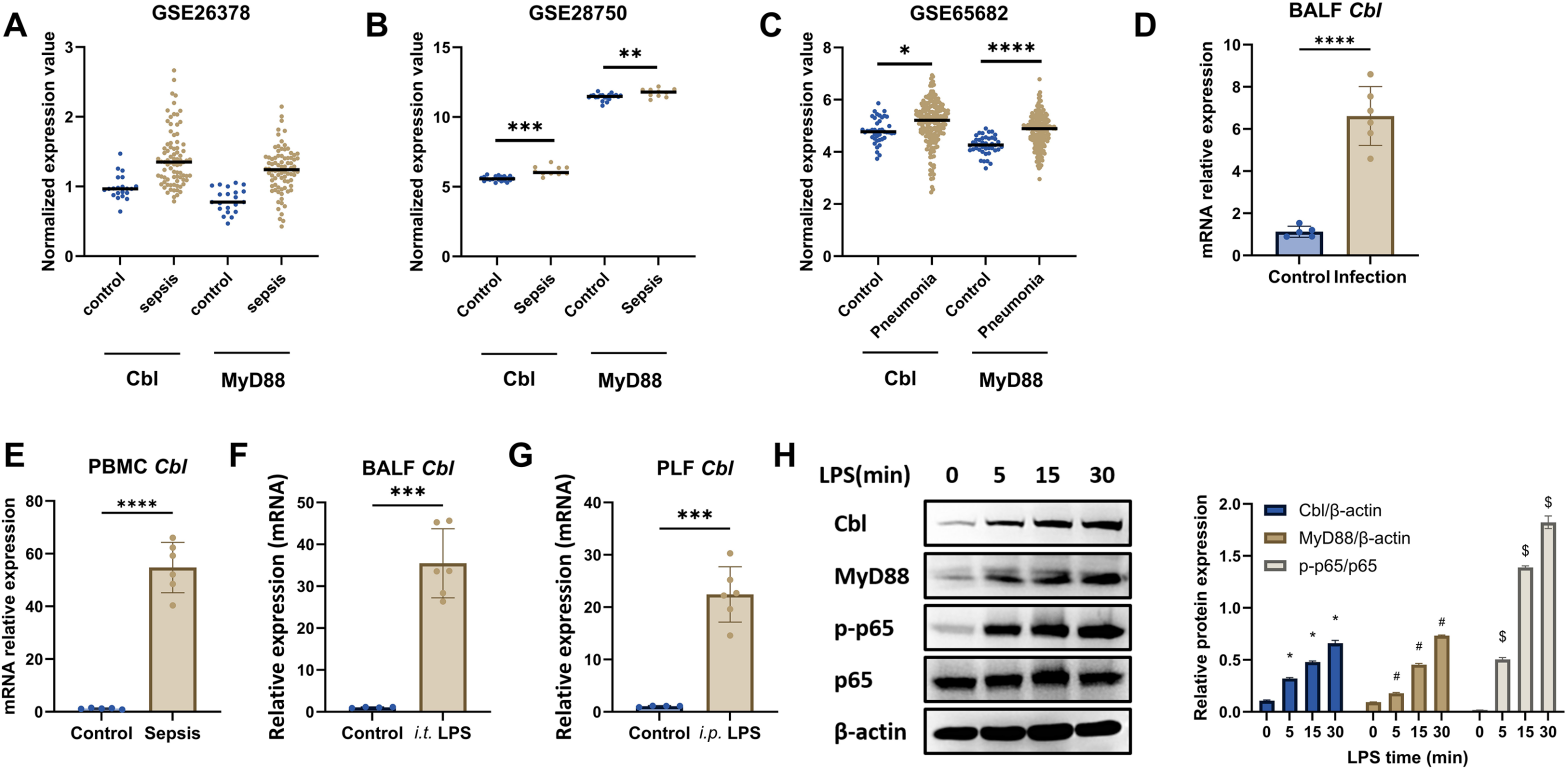
Cbl is upregulated in macrophages during inflammation. **(A–C)** Normalized transcriptional levels of Cbl and MyD88 in datasets of blood samples from sepsis patients or patients with pneumonia in the GEO database analyzed via GEO2R’s default limma pipeline (R package v3.48.3) (GSE26378, n=103; GSE28750, n=30; GSE65682, n=802); **(D, E)** mRNA level of Cbl in BALF from pneumonia patients (n=6) and nonpneumonia patients (n=5), as well as PBMCs from septic patients with abdominal or bloodstream infection (healthy control=5, sepsis=6, baseline in **Supplementary Tables S1**, **Supplementary Tables S2**). **(F, G)** mRNA level of Cbl in BALF from intratracheal LPS-challenged mice (n=6) and PLF from intraperitoneal LPS-challenged mice (n=6); **(H)** Western blot and quantification analysis of Cbl, MyD88, phospho-p65 and total p65 in BMDMs stimulated with LPS (100 ng/ml) at various time points; *p<0.05, **p<0.01, ***p<0.001, ****p<0.0001, #p<0.05 and $p<0.05 compared to 0 minutes group; Student’s t test; n=biological replicates.

To investigate the expression patterns of Cbl and MyD88 in clinical samples, we analyzed the transcriptional profiles of blood samples from three GSE datasets and found that Cbl, along with elevated MyD88 expression, was upregulated in both sepsis patients and pneumonia patients (**Figures 1A-C**). Our own cohort also confirmed this pattern. For patients with pneumonia, we collected bronchoalveolar lavage fluid (BALF) and isolated the cells. We found that the mRNA level of Cbl in BALF was greater than that in patients who underwent pulmonary nodule biopsy without pulmonary infection (**Figure 1D**) (baseline in **Supplementary Table S2**). Similar results were obtained from septic patients with abdominal or blood stream infection, in which Cbl mRNA in PBMCs was elevated compared with that in healthy controls (**Figure 1E**) (baseline in **Supplementary Table S3**). We also investigated the Cbl level in experimental animal models. In intratracheal LPS-induced acute lung injury, Cbl mRNA levels were increased in the BALF (**Figure 1F**). In the intraperitoneal LPS-injection model, the level of Cbl mRNA in the peritoneal lavage fluid (PLF) was also greater than that in the control group (**Figure 1G**). Thus, these results indicated that Cbl was upregulated during inflammation.

Since the above results were obtained from BALF, PLF or PBMCs derived from multicellular samples, we further analyzed the expression pattern of Cbl in macrophages. In LPS-stimulated BMDMs, the mRNA and protein levels were elevated (**Figure 1H**). Notably, Cbl was increased within five minutes of LPS stimulation, suggesting that Cbl is a fast-responding molecule during inflammation. Taken together, these results indicate that Cbl is upregulated in macrophages during inflammation.

### Myeloid deficiency of Cbl heightened inflammatory responses both *in vitro* and *in vivo*

**Figure 2:**
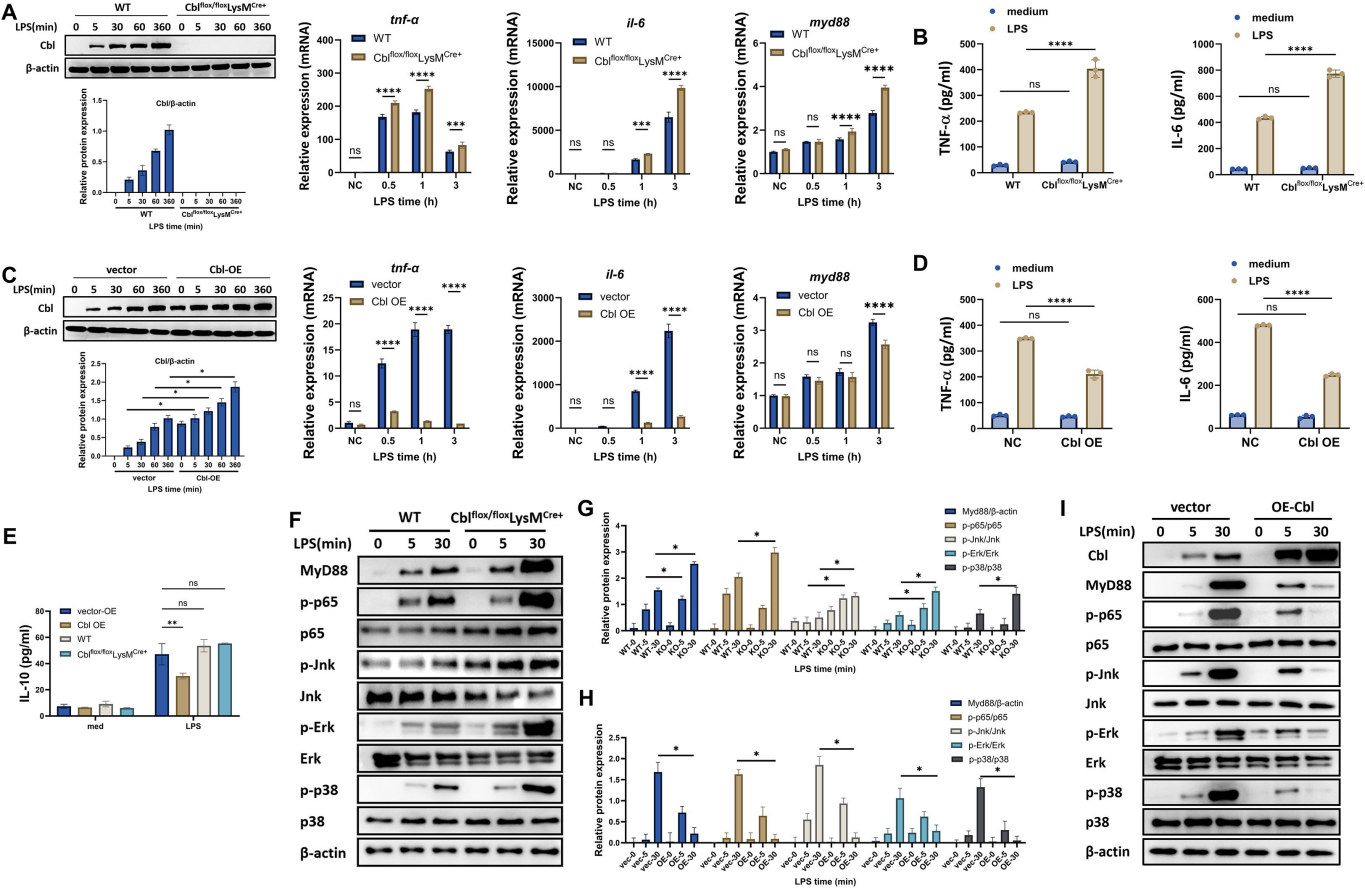
Myeloid deficiency of Cbl heightened inflammatory responses both *in vitro* and *in vivo*. **(A)** Western blot and quantification analysis of knockout efficiency of Cbl in BMDMs, and mRNA levels of tnf-α, il-6 and myd88 in BMDMs from Cbl^flox/flox^LysM^Cre+^ or wild-type mice stimulated with LPS (100 ng/ml) for the indicated durations (n=3); **(B)** Cytokine levels in the supernatants of BMDMs from Cbl^flox/flox^LysM^Cre+^ or wild-type mice stimulated with LPS (100 ng/ml) for 6 hours (n=3). **(C)** Western blot and quantification analysis of overexpression efficiency of Cbl in RAW264.7 cells, and mRNA levels of tnf-alpha, il-6 and myd88 in RAW264.7 cells transfected with Cbl-overexpressing plasmids stimulated with LPS (100 ng/ml) for the indicated durations (n=3); **(D)** Cytokine levels in the supernatant of RAW264.7 cells transfected with Cbl-overexpressing plasmid and stimulated with LPS (100 ng/ml) for 6 hours (n=3). **(E)** IL-10 levels in the supernatants of RAW264.7 cells transfected with Cbl-overexpressing plasmids or BMDMs from Cbl^flox/flox^LysM^Cre+^ or wild-type mice stimulated with LPS (100 ng/ml) for 6 hours (n=3). **(F, G)** Western blot and quantification analysis of the indicated molecules in BMDMs from Cbl^flox/flox^LysM^Cre+^ or wild-type mice stimulated with LPS (100 ng/ml) at various time points. **(H, I)** Western blot and quantification analysis of the indicated molecules in RAW264.7 cells transfected with Cbl-overexpressing plasmids stimulated with LPS (100 ng/ml) at various time points. *p<0.05, **p<0.01, ***p<0.001, ****p<0.0001, ns, not significant; two-way ANOVA; n=biological replicates.

To confirm the regulatory role of Cbl in macrophages, mice deficient in Cbl (Cbl^flox/flox^LysM^Cre+^) were generated, and BMDMs were prepared to analyze the regulation of Cbl in macrophages. We found that Cbl-deficient macrophages presented increased transcription of TNF-α and IL-6 at 0.5, 1 and 3 hours and MyD88 at 1 and 3 hours post-LPS stimulation (**Figure 2A**). Moreover, cytokine production in the supernatant six hours post-stimulation was also elevated in the deficient macrophages (**Figure 2B**). In contrast, in RAW264.7 cells transfected with Cbl-overexpressing plasmids, both the mRNA and protein levels of the above cytokines were suppressed, and the level of the MyD88 mRNA decreased only 3 hours post-stimulation (**Figures 2C, D**). These results indicated that Cbl deficiency upregulated the *de novo* synthesis of inflammatory cytokines, suggesting that Cbl may regulate signal transduction via the TLR4 pathway.

A previous study reported that Cbl could induce IL-10 production in DCs (17). To investigate the possible mechanism of Cbl in macrophages, we analyzed the IL-10 level in both Cbl-overexpressing RAW264.7 and Cbl-myeloid-deficient mice. We found that Cbl overexpression decreased IL-10 production, whereas Cbl deficiency did not alter the IL-10 level (**Figure 2E**), thus ruling out the involvement of IL-10 in the regulatory mechanism of Cbl in macrophages.

The increased mRNA and protein levels of inflammatory cytokines in Cbl-deficient macrophages suggest increased activation of TLR4-MyD88 signaling, which activates transcription factors, including phospho-p65 (p-p65), phospho-JNK (p-JNK), phospho-ERK (p-ERK) and phospho-p38 (p-p38). Therefore, we analyzed the involvement of Cbl in TLR4-MyD88 signaling. Our data revealed that Cbl deficiency upregulated MyD88 and p65, JNK, ERK and p38 phosphorylation (**Figures 2F, G**), whereas Cbl overexpression decreased the activation or phosphorylation of these molecules (**Figures 2H, I**). Thus, our data suggested that Cbl regulated inflammatory cytokines through TLR4-MyD88 signaling.

### Cbl promotes the ubiquitination-mediated degradation of MyD88 in macrophages

**Figure 3:**
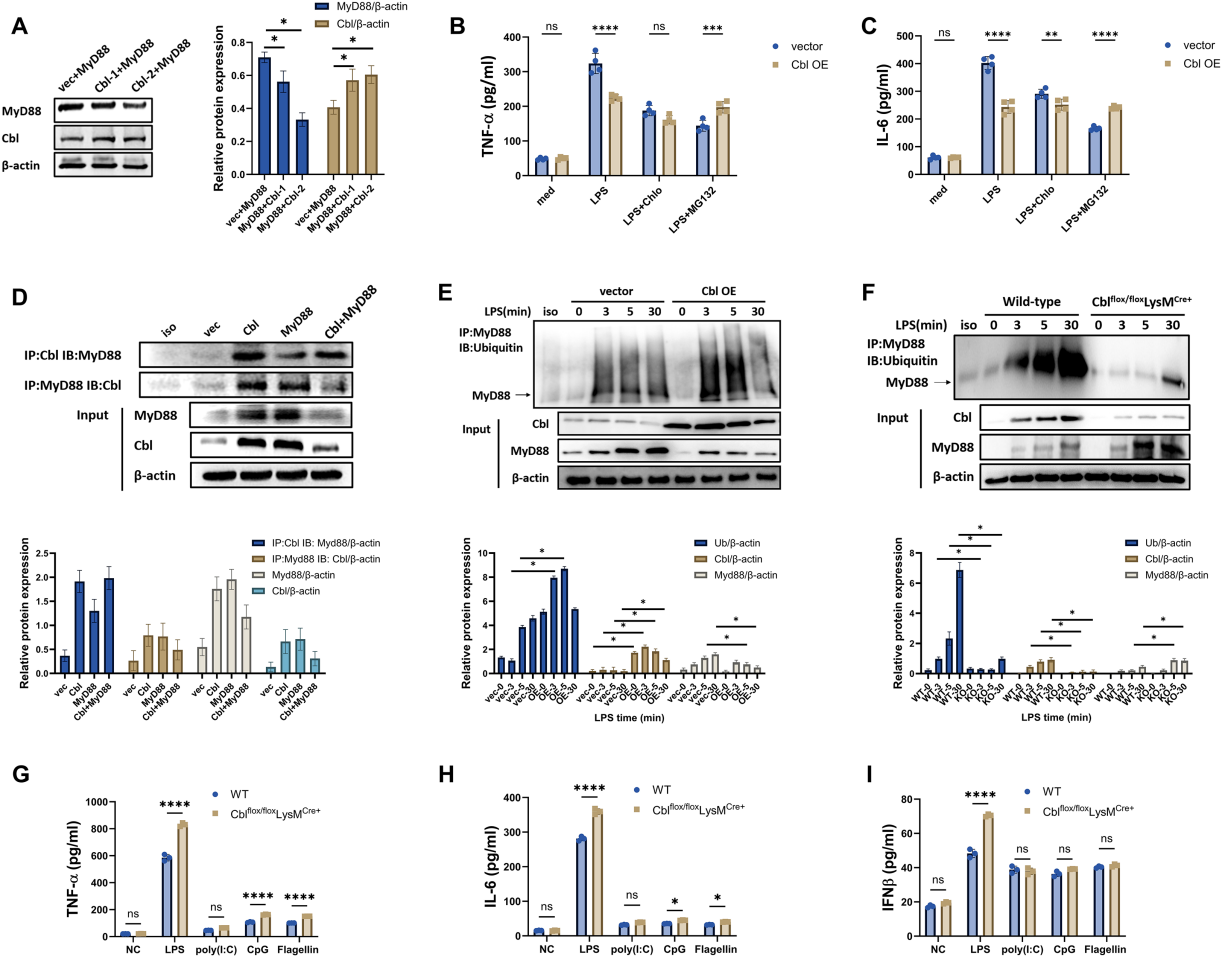
Cbl promotes the ubiquitination-mediated degradation of MyD88 in macrophages. **(A)** Western blot and quantification analysis of MyD88 and Cbl in RAW264.7 cells transfected with Cbl-overexpressing plasmids and transfected with elevated plasmid concentrations (Cbl-1 indicates 1 µg/ml plasmid; Cbl-2 indicates 2 µg/ml plasmid). **(B, C)** Cytokine levels in the supernatant of RAW264.7 cells transfected with Cbl-overexpressing plasmids, pretreated with MG-132 or chloroquine for 30 minutes and then stimulated with LPS (100 ng/ml) for 6 hours (n=4). **(D)** Immunoprecipitation and quantification analysis of MyD88 and Cbl in RAW264.7 cells transfected with Cbl- or MyD88-overexpressing plasmids; **(E, F)** Ubiquitination assay and quantification of MyD88 immunoprecipitated from RAW264.7 cells via Cbl-overexpressing plasmid transfection **(E)** or from BMDMs from Cbl^flox/flox^LysM^Cre+^ or wild-type mice **(F)** stimulated with LPS (100 ng/ml) for the indicated durations. **(G-I)** Cytokine levels in the supernatant of BMDMs from Cblflox/floxLysMCre+ or wild-type mice stimulated with LPS (100 ng/ml), poly(I:C) (5 μg/ml), CpG (0.2 μM) and Flagellin (1μM) for the six hours. ns, not statistically significant, *p<0.05, **p<0.01, ***p<0.001, ****p<0.0001; two-way ANOVA; n=biological replicates.

The downregulation of MyD88 by Cbl overexpression suggested possible degradation through ubiquitination, since Cbl is a ubiquitin E3 ligase. Indeed, cotransfection of Cbl and MyD88 dose-dependently downregulated MyD88 in HEK293 cells (**Figure 3A**). Protein degradation consists of proteasome- or lysosome-dependent pathways. We found that the attenuation of IL-6 and TNF-alpha in Cbl-overexpressing RAW264.7 cells was completely reversed when RAW264.7 cells were pretreated with MG132, a proteasome inhibitor, whereas the effects of chloroquine, an autophagy inhibitor, were only partially reversed (**Figures 3B, C**). Moreover, the immunoprecipitation results confirmed the mutual interaction between Cbl and MyD88 (**Figure 3D**). This result suggested that Cbl may promote the ubiquitin-mediated degradation of MyD88 in macrophages.

To prove the ubiquitination of MyD88, we performed ubiquitin analysis of MyD88 and found that the overexpression of Cbl promoted the ubiquitination of MyD88 (**Figure 3E**). Increased ubiquitin levels were observed at three and five minutes post-LPS stimulation (**Figure 3E**), whereas Cbl deficiency almost completely inhibited the ubiquitination of MyD88 (**Figure 3F**). Taken together, the present data indicates that Cbl promotes the ubiquitination and degradation of MyD88 in macrophages.

To further investigate the specificity of Cbl for MyD88 in other TLR signaling pathways, we stimulated BMDMs from Cbl^flox/flox^LysM^Cre+^ and WT mice with various TLR ligands that signal through MyD88-dependent and -independent mechanisms, including LPS (a ligand for TLR4), poly(I:C) (a ligand for TLR3), CpG (a ligand for TLR9), and flagellin (a ligand for TLR5), and measured the production of TNF-α, IL-6, and IFNβ. TLR5 and TLR9 recruits MyD88 as adaptor for signal, TLR3 recruits TRIF that independent of MyD88, and TLR4 recruits both for downstream pathways (18–20). As shown in **Figures 3G–I**, upon stimulation with LPS, CpG, or flagellin, Cbl^flox/flox^LysM^Cre+^ macrophages presented significant increases in TNF-α and IL-6 secretion compared with WT cells, whereas no significant difference was observed under poly(I:C) stimulation. These results suggest that MyD88 regulation by Cbl is conserved in multiple types of TLR signaling. For IFNβ, an inflammatory cytokine produced through a MyD88-independent mechanism, as shown in **Figure 3I**, only LPS stimulation induced a significant increase in Cbl^flox/flox^LysM^Cre+^ macrophages, whereas no differences were observed under poly(I:C), CpG, or flagellin stimulation between the two groups. Since a previous study indicated that overactivated MyD88 in TLR4 signaling could potentiate TBK1-mediated IFNβ production (19), these results collectively indicate that Cbl specifically regulates MyD88-dependent TLR signaling pathways, but not involved in TRIF-dependent signaling.

### Cbl directly regulates MyD88 ubiquitination independent of Cbl-b and Nedd4

**Figure 4:**
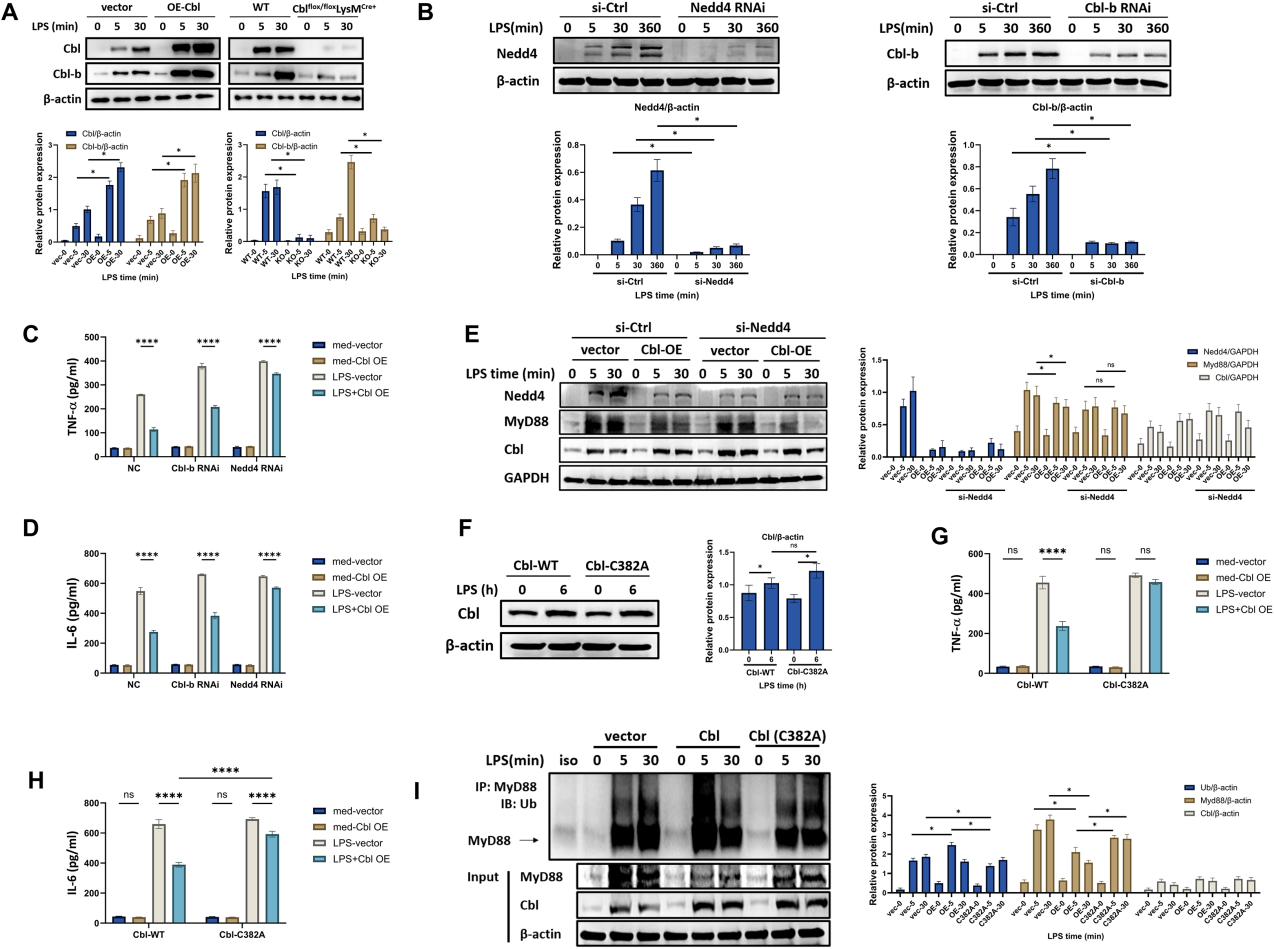
Cbl directly regulates MyD88 ubiquitination independent of Cbl-b and Nedd4. **(A)** Western blot and quantification analysis of Cbl and Cbl-b in RAW264.7 cells transfected with Cbl-overexpressing plasmids or BMDMs from Cbl^flox/flox^LysM^Cre+^ or wild-type mice stimulated with LPS (100 ng/ml) at various time points; **(B)** Western blot and quantification analysis of knockout efficiency of Nedd4 and Cbl-b in BMDMs stimulated with LPS (100 ng/ml) at various time points; **(C, D)** Cytokine levels in the supernatants of RAW264.7 cells transfected with Cbl-overexpressing plasmids and Cbl-b- or Nedd4-specific siRNAs, stimulated with LPS (100 ng/ml) for 6 hours (n=3). **(E)** Western blot and quantification analysis of Cbl and MyD88 in RAW264.7 cells transfected with Cbl-overexpressing plasmids and Nedd4-specific siRNAs and stimulated with LPS (100 ng/ml) for the indicated durations; **(F)** Western blot and quantification analysis of Cbl in RAW264.7 cells transfected with Cbl- or Cbl EF-hand-mutant (C382A) overexpressing plasmids and stimulated with LPS (100 ng/ml) for 6 hours. **(G, H)** Cytokine levels in the supernatant of RAW264.7 cells transfected with the Cbl-C382A-overexpressing plasmid and stimulated with LPS (100 ng/ml) for 6 hours (n=3). **(I)** Ubiquitination assay and quantification of immunoprecipitated MyD88 in RAW264.7 cells transfected with Cbl or Cbl-C382A–overexpressing plasmid and stimulated with LPS (100 ng/ml) for the indicated durations. ****p<0.0001, *p<0.05; two-way ANOVA; n=biological replicates.

Previous studies have demonstrated that Cbl-b can bind with MyD88 and recruit Nedd4 to induce the ubiquitination of MyD88 (21, 22). In this case, Cbl-b acts as a scaffold, and Nedd4 induces MyD88 ubiquitination as E3 ligase. Since Cbl shares a similar structure with Cbl-b, we asked whether Cbl-b or Nedd4 are involved in the regulatory mechanism of Cbl to MyD88. We found that Cbl overexpression indeed upregulated Cbl-b expression in RAW264.7 cells, whereas Cbl deficiency diminished Cbl-b expression in BMDMs (**Figure 4A**). However, Cbl-b knockdown had no effect on the attenuation of cytokine production caused by Cbl overexpression, indicating that Cbl regulated inflammation independent of Cbl-b (**Figures 4B, C**). In Nedd4-knockdown macrophages, although the effects of Cbl overexpression were not fully abrogated, Nedd4 knockdown partially reversed the Cbl-mediated suppression of cytokine production (**Figures 4B–D**). To confirm the regulatory role of Nedd4 in MyD88 in Cbl-overexpressing macrophages, we analyzed MyD88 and found that the overexpression of Cbl still led to the degradation of MyD88 (**Figure 4E**), suggesting that Cbl-induced ubiquitination was independent of Nedd4. To confirm the direct role of Cbl in MyD88 ubiquitination, we constructed a mutant, Cbl-C382A, whose RING Finger (RF) domain is defective and fails to induce ubiquitination of the target protein but is intact in protein interactions (17, 23). This mutation enabled us to investigate whether Cbl acts as a scaffold for Nedd4 or directly induces MyD88 ubiquitination. We found that Cbl-C382A overexpression could not alleviate cytokine production in macrophages, and the attenuation of TNF-alpha and IL-6 was largely compromised (**Figures 4F–H**). Moreover, the ubiquitination of MyD88 was inhibited in Cbl-C382A-mutant macrophages compared with wild-type Cbl-transfected macrophages (**Figure 4I**). The degradation of MyD88 was also abolished in Cbl-C382A-mutant macrophages (**Figure 4I**). Taken together, our results indicate that Cbl can directly induce MyD88 ubiquitination independent of Cbl-b and Nedd4.

### Cbl activation requires phosphorylation and calcium mobilization

**Figure 5:**
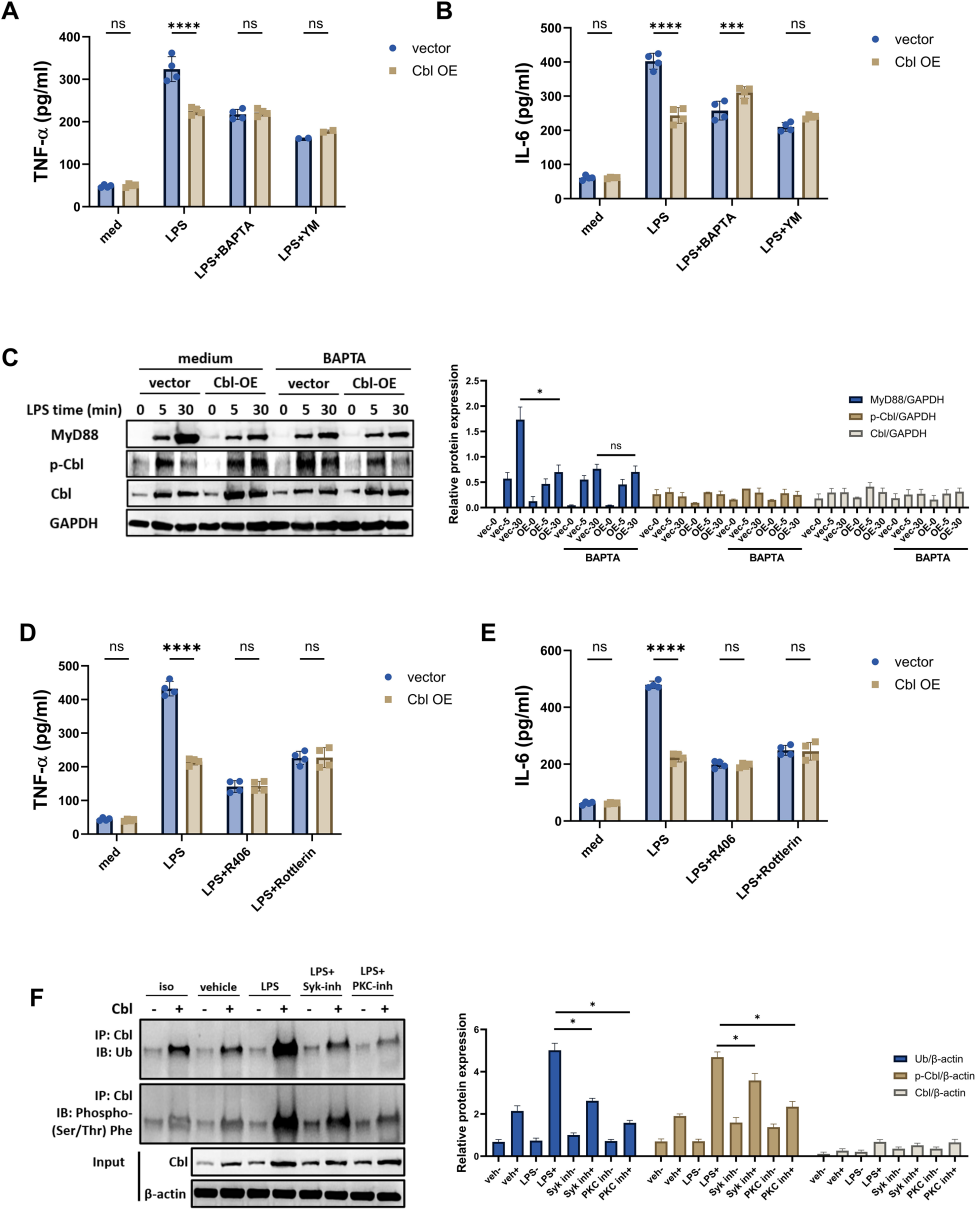
Cbl activation requires phosphorylation and calcium mobilization. **(A, B)** Cytokine levels in the supernatants of RAW264.7 cells transfected with Cbl-overexpressing plasmids, pretreated with BAPTA or YM for 30 minutes and then stimulated with LPS (100 ng/ml) for 6 hours (n=4). **(C)** Western blot and quantification analysis of phosphor-Cbl, Cbl and MyD88 in RAW264.7 cells transfected with Cbl-overexpressing plasmids, pretreated with BAPTA for 30 minutes and then stimulated with LPS (100 ng/ml) for the indicated durations; **(D, E)** Cytokine levels in the supernatant of RAW264.7 cells transfected with Cbl-overexpressing plasmid, pretreated with R406 or rottlerin for 30 minutes and then stimulated with LPS (100 ng/ml) for 6 hours (n=4). **(F)** Ubiquitination, phosphorylation and quantification analysis of immunoprecipitated Cbl in RAW264.7 cells transfected with Cbl-overexpressing plasmids, pretreated with the indicated inhibitors for 30 minutes, and stimulated with LPS (100 ng/ml) for the indicated durations. ns, not statistically significant; *p<0.05, ***p<0.001, ****p<0.0001; two-way ANOVA; n=biological replicates.

Structural analysis of Cbl suggested that the calcium-interacting capacity is based on the EF-hand domain (24, 25). We asked whether calcium is crucial for the ability of Cbl to regulate MyD88 in macrophages. We used two inhibitors of calcium signals, YM, an inhibitor of CRAC channels that inhibits store-operated calcium entry (SOCE), and BAPTA, a calcium chelator, to analyze the essential role of calcium in Cbl function (26, 27). We found, though calcium inhibition attenuated cytokine production in vector group that in accordance with previous published study (27), Cbl overexpression could not further reduce the cytokine production in the presence of both inhibitors in RAW264.7 cells (**Figures 5A, B**). Notably, calcium chelation also abrogated MyD88 degradation (**Figure 5C**). These results indicate that calcium mobilization is crucial for Cbl activation and subsequent MyD88 ubiquitination degradation.

Previous studies suggested the essential role of Cbl phosphorylation in ubiquitination and reported that Syk and PKC could phosphorylate Cbl (28–30). Since Syk and PKC can also be activated in LPS-stimulated macrophages, we asked whether Syk- or PKC-induced phosphorylation is essential for Cbl activation. Though Syk and PKC inhibition (through R406 and Rottlerin, respectively) partially attenuated cytokine production as reported, we found Cbl overexpression could not further reduce the cytokine production in macrophages abrogated the attenuation of cytokine production in Cbl-overexpressing macrophages (**Figures 5D, E**). Moreover, through direct phosphorylation analysis, we demonstrated that Syk and PKC inhibition diminished the phosphorylation of Cbl, as well as its ubiquitination (**Figure 5F**), suggesting that phosphor-activation was essential for Cbl-mediated ubiquitination. Thus, our data indicates that calcium mobilization and phosphorylation are crucial for Cbl-mediated MyD88 ubiquitination.

## Discussion

Our study demonstrated that Cbl is an intrinsic regulator of TLR-MyD88 signaling in macrophages through a phosphorylation- and calcium-dependent ubiquitination mechanism. This work expands the current understanding of immunoregulatory networks by identifying Cbl as a novel E3 ligase that directly targets MyD88, revealing previously unrecognized layers of posttranslational control in inflammatory responses.

Our data demonstrate that Cbl functions as a direct negative regulator of MyD88 stability in macrophages.​​ While the ubiquitin–proteasome system is known to fine-tune MyD88 activity, the repertoire of involved E3 ligases remains incomplete. Prior studies implicated Cbl-b as a scaffold recruiting Nedd4 for MyD88 degradation and Smurf/Spop as indirect regulators. Here, we provide conclusive evidence that Cbl itself acts as an E3 ligase for MyD88. We first showed that molecules interact between MyD88 and Cbl via coimmunoprecipitation and demonstrated that cotransfection induces the downregulation of MyD88. Functionally, Cbl overexpression promotes the polyubiquitination and proteasomal degradation of MyD88, whereas myeloid-specific Cbl deletion abolishes endogenous MyD88 ubiquitination. Moreover, a RING domain mutant (Cbl-C382A) lost its ability to ubiquitinate without disrupting MyD88 binding, confirming that Cbl’s catalytic function is indispensable for MyD88 degradation. Critically, this regulation occurs independently of Cbl-b or Nedd4. This distinguishes Cbl from known MyD88 regulators and establishes it as a primary E3 ligase in this pathway. Moreover, as TLR4, TLR5 and TLR9 all recruit MyD88 as adaptors to activate downstream molecules, while TLR3 recruits TRIF through a MyD88-independent mechanism, our data demonstrated that Cbl-mediated regulation of MyD88 is a general mechanism across multiple MyD88-dependent TLR signaling pathways but may not involve TRIF-dependent signaling.

We also reveal the dual activation requirements for Cbl’s E3 ligase activity—calcium mobilization and phosphorylation—linking innate immune signaling to ubiquitination dynamics.​​ Structural analysis suggests that Cbl contains an EF-hand domain potentially responsive to calcium fluxes. Our data functionally validated this finding by showing that calcium chelation (BAPTA) or SOCE inhibition (YM-58483) completely blocked Cbl-mediated MyD88 degradation and cytokine suppression (**Figure 5**). Notably, BAPTA, YM-58483 and MG-132 attenuated inflammatory cytokine production upon LPS stimulation. This is due to the calcium mobilization and ubiquitin-mediated degradation of IKKα/β, both of which are essential for cytokine production (27, 31, 32). Therefore, since Cbl overexpression did not further reduce cytokine levels, these results indicate that calcium and proteasome degradation are crucial for Cbl function. Notably, although MG-132 reversed cytokine attenuation in Cbl-overexpressing macrophages, chloroquine also partially reversed this effect. We speculate that this may be because the lysosomal pathway also serves as a complementary route and can affect the degradation of specific proteins, thereby influencing immune responses (33–35). However, our data indicated that the ubiquitin–proteasome system is the main pathway for degrading MyD88 in Cbl-mediated regulation.

Furthermore, we identified Syk and PKC as upstream kinases essential for Cbl activation, as their pharmacological inhibition prevents Cbl phosphorylation and subsequent MyD88 ubiquitination. This dual dependency creates a precise activation checkpoint: TLR4 engagement triggers IP3R-mediated ER calcium release and SOCE, which coincides with rapid Cbl upregulation (36). This spatiotemporal coupling suggests that the calcium surge may serve as a “molecular switch” for licensed Cbl to dampen MyD88 signaling, preventing detrimental inflammation.

​​Our findings indicate a crucial role of Cbl in macrophages beyond its previously described functions in dendritic cells or lymphocytes. However, further studies are still needed to address the role of Cbl in other myeloid-derived immune cells. On the other hand,​ while Cbl-b is recognized for its ability to suppress T/NK cell activation and Cbl promotes IL-10 production in DCs (7, 14), we show that in macrophages, Cbl directly constrains proinflammatory responses by targeting the core TLR adaptor MyD88. Notably, Cbl deficiency enhances rather than suppresses IL-10 in macrophages, excluding IL-10 as an intermediary in the anti-inflammatory mechanism of Cbl. This contrasts with DCs and underscores the context-dependent roles of Cbl across immune cell subsets.

Collectively, our work delineates the Cbl–MyD88 axis as a fundamental regulator of TLR signaling. By coupling calcium and phosphorylation sensors to MyD88 degradation, Cbl establishes a tunable feedback loop that dynamically calibrates inflammation intensity. Therapeutically, enhancing Cbl activity could represent a strategy to treat MyD88-driven pathologies (e.g., sepsis and autoimmune disorders), while its conditional activation mechanism minimizes the risk of immunosuppression.

## Data Availability

The raw data supporting the conclusions of this article will be made available by the authors, without undue reservation.

## References

[1] Tan Y Kagan JC . Innate Immune Signaling Organelles Display Natural and Programmable Signaling Flexibility. Cell. (2019) 177:384–398.e311. doi: 10.1016/j.cell.2019.01.039, PMID: 30853218 PMC6710629

[2] Kawai T Ikegawa M Ori D Akira S . Decoding Toll-like receptors: Recent insights and perspectives in innate immunity. Immunity. (2024) 57:649–73. doi: 10.1016/j.immuni.2024.03.004, PMID: 38599164

[3] Takeuchi O Akira S . Pattern Recognition Receptors and Inflammation. Cell. (2010) 140:805–20. doi: 10.1016/j.cell.2010.01.022, PMID: 20303872

[4] Fitzgerald KA Kagan JC . Toll-like Receptors and the Control of Immunity. Cell. (2020) 180:1044–66. doi: 10.1016/j.cell.2020.02.041, PMID: 32164908 PMC9358771

[5] Lee SM Suk K Lee WH . Synthetic peptides containing ITIM-like sequences of IREM-1 (CD300F) differentially regulate MyD88 and TRIF-mediated TLR signalling through activation of SHP and/or PI3K. Clin Exp Immunol. (2012) 167:438–46. doi: 10.1111/j.1365-2249.2011.04528.x, PMID: 22288587 PMC3374276

[6] Gurung P Fan G Lukens JR Vogel P Tonks NK Kanneganti T-D . Tyrosine Kinase SYK Licenses MyD88 Adaptor Protein to Instigate IL-1α-Mediated Inflammatory Disease. Immunity. (2017) 46:635–48. doi: 10.1016/j.immuni.2017.03.014, PMID: 28410990 PMC5501252

[7] Han C Jin J Xu S Liu H Li N Cao X . Integrin CD11b negatively regulates TLR-triggered inflammatory responses by activating Syk promoting degradation MyD88 TRIF via Cbl-b. Nat Immunol. (2010) 11:734–42. doi: 10.1038/ni.1908, PMID: 20639876

[8] Wirnsberger G Zwolanek F Asaoka T Kozieradzki I Tortola L Wimmer RA . Inhibition of CBLB protects from lethal Candida albicans sepsis. Nat Med. (2016) 22:915–23. doi: 10.1038/nm.4134, PMID: 27428901 PMC6209141

[9] Budroni V Versteeg GA . Negative Regulation of the Innate Immune Response through Proteasomal Degradation and Deubiquitination. Viruses. (2021) 13. doi: 10.3390/v13040584, PMID: 33808506 PMC8066222

[10] Tang R Langdon WY Zhang J . Regulation of immune responses by E3 ubiquitin ligase Cbl-b. (2019). doi: 10.1016/j.cellimm.2018.11.002, PMID: 30442330 PMC6504630

[11] Henry CM Castellanos CA Buck MD Giampazolias E Frederico B Cardoso A . SYK ubiquitination by CBL E3 ligases restrains cross-presentation of dead cell-associated antigens by type 1 dendritic cells. Cell Rep. (2023), 42. doi: 10.1016/j.celrep.2023.113506, PMID: 38019655

[12] Tang R Langdon WY Zhang J . Negative regulation of receptor tyrosine kinases by ubiquitination: Key roles of the Cbl family of E3 ubiquitin ligases. Front Endocrinol. (2022) 13:971162. doi: 10.3389/fendo.2022.971162, PMID: 35966060 PMC9365936

[13] Yasuda T Tezuka T Maeda A Inazu T Yamanashi Y Gu H . Cbl-b Positively Regulates Btk-mediated Activation of Phospholipase C-γ2 in B Cells. J Exp Med. (2002) 196:51–63. doi: 10.1084/jem.20020068, PMID: 12093870 PMC2194016

[14] Zhu L-L Luo T-M Xu X Guo Y-H Zhao X-Q Wang T-T . E3 ubiquitin ligase Cbl-b negatively regulates C-type lectin receptor–mediated antifungal innate immunity. J Exp Med. (2016) 213:1555–70. doi: 10.1084/jem.20151932, PMID: 27432944 PMC4986534

[15] Zhang J Chiang YJ Hodes RJ Siraganian RP . Inactivation of c-Cbl or Cbl-b Differentially Affects Signaling from the High Affinity IgE Receptor. J Immunol. (2004) 173:1811–8. doi: 10.4049/jimmunol.173.3.1811, PMID: 15265912

[16] Yang Y-H Xie K-F Yang S Wang H Ma H-H Zhou M . BLNK negatively regulates innate antifungal immunity through inhibiting c-Cbl-mediated macrophage migration. Proc Natl Acad Sci. (2024) 121. doi: 10.1073/pnas.2400920121, PMID: 39413134 PMC11513953

[17] Duan J-L He H-Q Yu Y Liu T Ma S-J Li F . E3 ligase c-Cbl regulates intestinal inflammation through suppressing fungi-induced noncanonical NF-κB activation. Sci Adv. (2021) 7. doi: 10.1126/sciadv.abe5171, PMID: 33962939 PMC8104877

[18] Cai S Batra S Shen L Wakamatsu N Jeyaseelan S . Both TRIF- and MyD88-Dependent Signaling Contribute to Host Defense against Pulmonary Klebsiella Infection. J Immunol. (2009) 183:6629–38. doi: 10.4049/jimmunol.0901033, PMID: 19846873 PMC2777750

[19] Zhou J Sun T Jin S Guo Z Cui J . Dual Feedforward Loops Modulate Type I Interferon Responses and Induce Selective Gene Expression during TLR4 Activation. iScience. (2020) 23:100881. doi: 10.1016/j.isci.2020.100881, PMID: 32062450 PMC7021547

[20] Fitzgerald KA Rowe DC Barnes BJ Caffrey DR Visintin A Latz E . LPS-TLR4 Signaling to IRF-3/7 and NF-κB Involves the Toll Adapters TRAM and TRIF. J Exp Med. (2003) 198:1043–55. doi: 10.1084/jem.20031023, PMID: 14517278 PMC2194210

[21] Magnifico A Ettenberg S Yang C Mariano J Tiwari S Fang S . WW domain HECT E3s target Cbl RING finger E3s for proteasomal degradation. J Biol Chem. (2003) 278:43169–77. doi: 10.1074/jbc.M308009200, PMID: 12907674

[22] Zhang Q Lee W-B Kang J-S Kim LK Kim Y-J . Integrin CD11b negatively regulates Mincle-induced Signaling via Lyn–SIRPα–SHP1 complex. Exp Mol Med. (2018) 50:e439–9. doi: 10.1038/emm.2017.256, PMID: 29400702 PMC5992981

[23] Levkowitz G Waterman H Ettenberg SA Katz M Tsygankov AY Alroy I . Ubiquitin Ligase Activity and Tyrosine Phosphorylation Underlie Suppression of Growth Factor Signaling by c-Cbl/Sli-1. Mol Cell. (1999) 4:1029–40. doi: 10.1016/s1097-2765(00)80231-2, PMID: 10635327

[24] Hu J Hubbard SR . Structural Characterization of a Novel Cbl Phosphotyrosine Recognition Motif in the APS Family of Adapter Proteins. J Biol Chem. (2005) 280:18943–9. doi: 10.1074/jbc.M414157200, PMID: 15737992

[25] Meng W Sawasdikosol S Burakoff SJ Eck MJ . ). Structure of the amino-terminal domain of Cbl complexed to its binding site on ZAP-70 kinase. Nature. (1999) 398:84–90. doi: 10.1038/18050, PMID: 10078535

[26] Schappe MS Szteyn K Stremska ME Mendu SK Downs TK Seegren PV . Chanzyme TRPM7 Mediates the Ca2+ Influx Essential for Lipopolysaccharide-Induced Toll-Like Receptor 4 Endocytosis and Macrophage Activation. Immunity. (2018) 48:59–74.e55. doi: 10.1016/j.immuni.2017.11.026, PMID: 29343440 PMC5783319

[27] Fu Z Wang D Zheng C Xie M Chen Y Zhou Y . Elimination of intracellular Ca(2+) overload by BAPTA−AM liposome nanoparticles: A promising treatment for acute pancreatitis. Int J Mol Med. (2024) 53. doi: 10.3892/ijmm.2024.5358, PMID: 38390952 PMC10903929

[28] Meyer RD Husain D Rahimi N . c-Cbl inhibits angiogenesis and tumor growth by suppressing activation of PLCγ1. Oncogene. (2011) 30:2198–206. doi: 10.1038/onc.2010.597, PMID: 21242968 PMC3969724

[29] Swaminathan G Tsygankov AY . The Cbl family proteins: Ring leaders in regulation of cell signaling. J Cell Physiol. (2006) 209:21–43. doi: 10.1002/jcp.20694, PMID: 16741904

[30] Yasuda T Maeda A Kurosaki M Tezuka T Hironaka K Yamamoto T . Cbl Suppresses B Cell Receptor–Mediated Phospholipase C (Plc)-γ2 Activation by Regulating B Cell Linker Protein–Plc-γ2 Binding. J Exp Med. (2000) 191:641–50. doi: 10.1084/jem.191.4.641, PMID: 10684856 PMC2195830

[31] Fu Z Fan Q Zhou Y Zhao Y He Z . Elimination of Intracellular Calcium Overload by BAPTA-AM-Loaded Liposomes: A Promising Therapeutic Agent for Acute Liver Failure. ACS Appl Mater Interfaces. (2019) 11:39574–85. doi: 10.1021/acsami.9b13690, PMID: 31589019

[32] Tiwari RL Singh V Singh A Rana M Verma A Kothari N . PKCδ-IRAK1 axis regulates oxidized LDL-induced IL-1β production in monocytes. J Lipid Res. (2014) 55:1226–44. doi: 10.1194/jlr.M045658, PMID: 24792928 PMC4076070

[33] Li Q Wang F Wang Q Zhang N Zheng J Zheng M . SPOP promotes ubiquitination and degradation of MyD88 to suppress the innate immune response. PloS Pathog. (2020) 16:e1008188. doi: 10.1371/journal.ppat.1008188, PMID: 32365080 PMC7224567

[34] Wang J Maldonado MA . The ubiquitin-proteasome system and its role in inflammatory and autoimmune diseases. Cell Mol Immunol. (2006) 3:255–61. 16978533

[35] Ma S Attarwala IY Xie XQ . SQSTM1/p62: A Potential Target for Neurodegenerative Disease. ACS Chem Neurosci. (2019) 10:2094–114. doi: 10.1021/acschemneuro.8b00516, PMID: 30657305 PMC6712989

[36] Mukherjee R Das A Chakrabarti S Chakrabarti O . Calcium dependent regulation of protein ubiquitination – Interplay between E3 ligases and calcium binding proteins. Biochim Biophys Acta (BBA) - Mol Cell Res. (2017) 1864:1227–35. doi: 10.1016/j.bbamcr.2017.03.001, PMID: 28285986

