# Supplementary material for: Cbl promotes MyD88 ubiquitin-mediated degradation in macrophages via phosphorylation and calcium mobilization
Source: Front Immunol. 2026 Jan 5;16:1679035. doi: 10.3389/fimmu.2025.1679035 (PMC12812934; doi:10.3389/fimmu.2025.1679035)
Supplement: Supplementary file 1 [file Table1.docx]

**Supplementary Table S1.** The baseline of pneumonia patients and non-pneumonia patients included in the study.

|  | **Non-Pneumonia (n=5)** | **Pneumonia (n=6)** | ***P*** |
| --- | --- | --- | --- |
| **Basic Characteristics** | | | |
| Age, years | 56 (50–62) | 58 (52–65) | 0.618 |
| Weight, kg | 67 (61–72) | 64 (58–70) | 0.473 |
| Sex, male | 3 (60.0%) | 4 (66.7%) | 0.836 |
| **Laboratory tests** | | | |
| Hemoglobin, g/L | 138 (130–145) | 126 (118–135) | 0.089 |
| White blood cell count, ×10⁹/L | 5.9 (5.2–6.5) | 12.8 (10.5–15.2) | <0.001 |
| Neutrophil count, ×10⁹/L | 3.6 (3.0–4.1) | 9.7 (8.2–11.5) | <0.001 |
| Platelet count, ×10⁹/L | 245 (218–270) | 202 (165–235) | 0.124 |
| Albumin, g/L | 43 (41–45) | 35 (32–38) | 0.001 |
| Total bilirubin, μmol/L | 9.8 (8.0–11.5) | 15.2 (12.0–18.5) | 0.012 |
| ALT, U/L | 22 (17–26) | 38 (28–48) | 0.015 |
| AST, U/L | 20 (16–24) | 36 (29–45) | 0.009 |

Notes: Data presented as number (percentage) or median (interquartile range). ALT: Alanine aminotransferase; AST: Aspartate aminotransferase; PCT: Procalcitonin; *P*-value is calculated by t-test, Mann-Whitney U test or Fisher's exact test as appropriate.

**Supplementary Table S2.** The baseline of healthy controls and septic patients included in the study.

|  | **Healthy Control (n=5)** | **Sepsis (n=6)** | ***P*** |
| --- | --- | --- | --- |
| **Basic Characteristics** | | | |
| Age, years | 52 (45–58) | 55 (48–62) | 0.482 |
| Weight, kg | 68 (62–73) | 65 (59–70) | 0.531 |
| Sex, male | 3 (60.0%) | 4 (66.7%) | 0.836 |
| **Laboratory tests** | | | |
| Hemoglobin, g/L | 135 (128–142) | 128 (115–136) | 0.217 |
| White blood cell count, ×10⁹/L | 6.2 (5.5–6.8) | 15.8 (12.3–18.5) | <0.001 |
| Neutrophil count, ×10⁹/L | 3.8 (3.2–4.3) | 12.6 (10.1–14.8) | <0.001 |
| Platelet count, ×10⁹/L | 252 (225–280) | 185 (142–220) | 0.052 |
| Albumin, g/L | 42 (40–44) | 32 (29–35) | 0.002 |
| Total bilirubin, μmol/L | 10.2 (8.5–12.0) | 18.5 (14.3–22.8) | 0.006 |
| ALT, U/L | 23 (18–28) | 45 (32–58) | 0.008 |
| AST, U/L | 21 (17–25) | 42 (30–55) | 0.007 |

Notes: Data presented as number (percentage) or median (interquartile range). ALT: Alanine aminotransferase; AST: Aspartate aminotransferase; PCT: Procalcitonin; *P*-value is calculated by t-test, Mann-Whitney U test or Fisher's exact test as appropriate.
